# Initial response to SARS-CoV-2 (COVID-19) outbreak in Sri Lanka; views of public health specialists through an International Health Regulations lens

**DOI:** 10.1371/journal.pone.0293521

**Published:** 2023-11-10

**Authors:** Amandhi Caldera, Rajitha Wickremasinghe, Gretchen Newby, Ruwanthi Perera, Kamini Mendis, Deepika Fernando

**Affiliations:** 1 Department of Public Health, Faculty of Medicine, University of Kelaniya, Kelaniya, Sri Lanka; 2 Malaria Elimination Initiative, Institute for Global Health Sciences, University of California, San Francisco, San Francisco, CA, United States of America; 3 Department of Parasitology, Faculty of Medicine, University of Colombo, Colombo, Sri Lanka; Freelance Consultant, Myanmar, MYANMAR

## Abstract

The COVID-19 pandemic affected Sri Lanka despite having developed an International Health Regulations (IHR) steering committee in 2016 and a national action plan for health security following the Joint External Evaluation in 2018. Many steps were taken to improve the disaster management skills of healthcare workers even before the COVID-19 outbreak. We interviewed seven public health specialists to obtain their views on the country’s response to the pandemic. A thematic analysis was conducted, leading to the emergence of three major themes and seven subthemes. The major themes included health security preparedness; COVID-19 management; and effects of COVID-19. The subthemes were; preparedness prior to pandemic and gaps in the preparedness (under health security preparedness); dual burden for the curative sector, strategies to reduce transmission and barriers to managing COVID-19 (under COVID-19 management) and negative and positive effects of COVID-19 (under effects of COVID-19). When COVID-19 reached Sri Lanka, healthcare workers, border control authorities and those involved with infectious disease control were overwhelmed by the magnitude of the pandemic. Healthcare workers’ hesitation to work amidst the pandemic due to fear of infection and possible transmission of infection to their families was a major issue; the demand for personal protective equipment by health workers when stocks were low was also a contributory factor. Lockdowns with curfew and quarantine at government regulated centers were implemented as necessary. Perceptions of the public including permitting healthcare workers to perform field public health services, logistical barriers and lack of human resources were a few of the barriers that were expressed. Some persons did not declare their symptoms, fearing that they would have to be quarantined. The pandemic severely affected the economy and Sri Lanka relied on donations and loans to overcome the situation. Pandemic also brought about innovative methods to maintain and upgrade health service provision. Individuals with non-communicable diseases received their regular medications through the post which reduced their risk of being infected by visiting hospitals. Improvement of laboratory services and quarantine services, a reduction of acute respiratory infections and diarrhoeal diseases, improved intersectoral coordination and public philanthropic response were other positive effects.

## Introduction

The International Health Regulations (IHR) is a legal framework to assist the international community to prevent or respond to acute public health risks. The responsibility of implementing IHR at national level lies with governments and all countries are required to have the ability to detect, assess and report and respond to public health risks and emergencies thereby limiting the spread of health risks to neighboring countries through travel and trade. In compliance with IHR, International Health Regulations are implemented in the Sri Lanka through the Quarantine Unit of the Ministry of Health with its offices located at ports of entry. Sri Lanka, a South Asian country, has a population of approximately 21.5 million. Over half of the population is concentrated in the Western, Central and Southern provinces which jointly covers less than one fourth of the total land area of the country. Government’s spending on health was 5.92% of the total government expenditure [1].

Sri Lanka took active steps prior to the pandemic to strengthen preparedness against a potential threat towards public health. Following the establishment of the IHR steering committee in Sri Lanka in 2016, the Ministry of Health and the World Health Organization (WHO) continued to take active steps to ensure preparedness. A five-year National Action Plan for Health Security was developed following the Joint External Evaluation in 2018. There were many steps taken to improve the skills of healthcare workers on disaster management even before the COVID-19 outbreak. In addition, training of security personnel at points of entry and securing a supply of Personal Protective Equipment (PPE) were steps taken towards preparedness prior to the pandemic. The Ministry of Health also had a pandemic preparedness plan that specifically focused on preparing for pandemics. Despite these efforts, however, there were gaps in the preparedness plan that were identified due to the overwhelming demands of the pandemic that put an enormous strain on the Sri Lanka’s health system.

The 2019 novel coronavirus, or severe acute respiratory syndrome coronavirus 2 (SARS-CoV-2), known as COVID-19, has rapidly spread from its origin in Wuhan City of China’s Hubei Province to the rest of the world [2]; China reported the first case of COVID-19 in response to abide by International Health Regulations. Since the origin of the virus, it has spread across the world with the emergence of new variants having varying degrees of virulence and transmissibility in many waves with more than 475 million cases and over 6 million deaths been reported by end March 2022 (https://www.worldometers.info/coronavirus/#countries).

The first confirmed COVID-19 case was reported in Sri Lanka on the 27^th^ January 2020 in a 44-year-old Chinese national from Hubei province, China. On 10^th^ March 2020, with the first local case being reported from a 52-year-old tour guide working with a group of Italian tourists the country went into an extremely stringent, curfew-type lockdown that imposed complete restrictions on people’s movements and prohibited social gatherings from 17^th^ March 2020 [3]. A COVID-19 task force was immediately established under the leadership of the country’s President which consisted of multidisciplinary experts such as epidemiologists, medical administrators, infectious diseases specialists, military personnel, police officers, social workers, politicians and media personnel. The military played a leading role in responding to the crisis, from overseeing quarantine centers to contact tracing, while the police managed the curfew [3].

Upon intensification of the health crisis, the government implemented further restrictions, including a temporary suspension of all inbound flights and on-arrival visas, mandatory quarantine of travelers arriving from high-risk countries in special quarantine centers, regular disinfection of market places and public transport stations, and wearing of face masks and sanitizing or handwashing when entering any facility, all of which contributed to the successful reduction of the disease transmission rate over the initial months of the epidemic by mid-September 2020 [3].

Since the inception of the epidemic in Sri Lanka in March 2020, three waves of infections have originated from six distinct clusters which erupted in different parts of the country although some of the clusters no longer exist with the emergence of new variants. Because of its commitment to preparedness under the IHR, Sri Lanka was relatively successful in controlling the first wave, from March to October 2020. The second wave, from October 2020 to April 2021, took a much longer time to control, probably due to control measures not being as stringent as during the first wave. The Sinhala New Year celebration in mid-April 2021, during which many travelled to their homes, led to the third wave of the epidemic which was not investigated in this study as the third wave was in progression at the time of our interviewing public health specialists. The aim of this study was to understand the views of public health specialists on COVID-19 control in Sri Lanka during the second wave of the infection in the country which was highly commended as part of a broader investigation of the effect of the pandemic on prevention of re-establishment of malaria in Sri Lanka.

## Materials and methods

This qualitative study was carried out in two phases; a key informant interview guide schedule addressing the research questions was developed and interviews were conducted during the first phase. In the second phase, the interview transcripts underwent thematic analysis. Patterns were identified through a rigorous process of data familiarization, data coding, and theme development and revision. Themes were derived after identifying patterns of meaning across the transcribed interviews. The study was carried out between November 2021 and March 2022. Ethics clearance was obtained from the Ethics Review Committee of the Faculty of Medicine, University of Kelaniya, Sri Lanka (ref. No: P/201/12/2021). The details of each phase are given below.

### Phase 1

#### Interview guide development

The semi structured interview guide was developed, reviewed, and edited by the research team. The interview guide was then tailored to reflect the specific role and expertise of each key informant to better capture their unique perspectives and experiences with COVID-19.

#### Participant identification and recruitment

Through a purposive sampling approach, as described by Green and Thorogood (2018), representatives of seven organizations that were intensely involved with COVID-19 management were selected as interviewees [4]. Seven key informant interviews were conducted from November, 2021 to March, 2023 comprising four Directors of units of the Ministry of Health, two specialists from international organizations and a Deputy Regional Director of Health Services who was on the ground managing and directing healthcare services, all of whom were medical doctors who were public health specialists.

#### Data collection

The identified key informants were contacted via phone, their cooperation sought and a suitable time for the interview via zoom was obtained. All key informants were mailed the participant information sheet. All key informant interviews were conducted over zoom; at the commencement of the interview, verbal consent to conduct the interview and permission to record the interview were obtained by the principal investigator. The principal investigator and another investigator were the interviewers in all key informant interviews. The research assistant was also present at all the interviews. Each interview was approximately 40 minutes in length.

### Phase 2

#### Interview transcription

After the research assistant listened to the interviews conducted in English for the purpose of familiarization, the interviews were transcribed. Almost all the interviews contained a few short sections of conversation that were not related to the interview questions and these sections of the interview were not included in the transcribed document. Furthermore, fillers in speech (e.g.- ummm, ugh…ah) and false starts were also not included in the transcribed and translated document unless it was essential to the context of what was stated.

On average, a 40-minute recording required about 3 hours to be transcribed. The transcribed interviews were approximately 6–8 single spaced pages in length.

#### Process of thematic analysis

The transcribed interviews were analyzed using Thematic Analysis described by Braun and Clark (2006) [5]. The six phases of thematic analysis were carried out to arrive at the themes. During the initial stage of the thematic analysis process, the research assistant familiarized herself with the data by reading the transcribed data 1–2 times. Preliminary codes were identified, paying close attention to recurring ideas in the interviews. These recurring ideas comprised themes. The themes were closely examined by the research assistant and the principal investigator to ensure that the responses were categorized under the most appropriate theme. Finally, sub-themes were identified within the main themes.

## Results and discussion

Three prominent themes were derived from the transcribed data. A summary of themes and the sub themes are included in Table 1.

**Table 1:** Summary of themes and subthemes.

Theme	Subtheme
Health security preparedness	• Preparedness prior to pandemic
	• Gaps in preparedness
COVID-19 management	• Dual burden for the curative sector
	• Strategies to reduce transmission
	• Barriers to managing COVID-19
Effects of COVID-19	• Negative effects
	• Positive effects

### Theme 1: Health security preparedness

#### Subtheme 1: Preparedness prior to pandemic

Representatives of the Ministry of Health and international organizations had several insights regarding the steps taken to strengthen preparedness prior to the pandemic based on the National Action Plan for Health Security. However, the preparedness plan that existed could not cope with the COVID-19 pandemic.

*“We were prepared for a similar outbreak at any time… through the disaster unit, we organized preparedness awareness programs focused on CBRNE (Chemical, Biological, Radiological, Nuclear, and Explosive) management…At trainings we spoke mainly about how to manage chemical and biological hazards … We also have a disaster preparedness diploma program … we strengthened the capacity of health workers especially at the National Institute of Infectious Diseases, Infectious Diseases Hospital, National Hospital and several other places so they will be able to handle some kind of biological hazard.”*

*(Director from Ministry of Health)*

*“We also trained the security forces to handle them* (chemical and biological hazards). *we provided initial supply of PPEs and similar items for those places…so therefore*, *when COVID hit*, *the initial procurement of such items was not a major issue*.

*(Director from Ministry of Health)*

*“it was only a just nominal services that we were having.. We had the pandemic influenza preparedness plan when the COVID-19 came, I think that helped a lot to face this pandemic.”*

*(Director from Ministry of Health)*

*“The ministry also had a specific pandemic preparedness plan which was done in 2012… that was developed after the SARS, MERS and the other influenza epidemics we have been experiencing globally… So, under that, we also meet once a month as a part of this influenza and preparedness plan”*

*(Representative from an international organization)*

After COVID-19 started spreading globally, the Ministry of Health along with the WHO took active steps to ensure monitoring of different viral strains coming through ports of entry into Sri Lanka.

*“In the meantime, luckily on the 2nd of January 2020 we had this influenza meeting and we tabled the COVID matter with all the virologists and at that time it was identified as a zoonotic disease in Wuhan and not much of a threat to global issues. But still we activated the pandemic preparedness plan so that we make sure that the points of entries are checked and to ensure that we don’t have any of these viral strains coming through the border… because that’s the only way that viruses can come to the country… Sri Lanka took a proactive role to control the threat”*

*(Representative from an international organization)*

#### Subtheme 2: Gaps in preparedness

Although the Ministry of Health and other organizations continuously worked in line with the National Action Plan for Health Security, when the pandemic hit Sri Lanka, the healthcare workers, border control authorities and those involved with infectious disease control were overwhelmed by the magnitude of the COVID-19 pandemic.

*“We never expected a condition like this. That means to this magnitude … no no…no way… Because of that, there were so many gaps we had… so we had to start from the beginning … and the disease condition was entirely different to the other things we had encountered or prepared for and rapidly spreading.”*

*(Director from Ministry of Health)*

*“Now if you look at that plan, our maximum thinking was influenza level and dengue. We never thought of a pandemic of this magnitude globally and that is why we struggled… not only developing countries, but developed countries are also struggling too.”*

*(Representative from an international organization)*

The Ministry of Health identified many gaps in the preparedness plan that mainly occurred because of the high demand for services that COVID-19 exerted on the health system. One of the main areas of difficulties was the lack of preparedness of health care workers to work amidst the pandemic. Interviewees spoke about the general fear of the COVID-19 pandemic among healthcare workers and how that affected d the commitment to their work. This, in turn, affected the efficiency of management during the early stages of the pandemic. The staff were demanding personal protective equipment which were in short supply. There was also misuse of personal protective equipment due to lack of knowledge of their use initially.

*“The health system was overwhelmed. So, we had to dedicate wards, and on top of that, entire hospitals were closed for other services…other health services were compromised”*

*(Director from Ministry of Health)*

*“Even though people were competent to handle the situation, most of our people were not ready to handle this situation. They were running. For instance, I had trained people in disaster management where you are supposed to go to the frontline in case an emergency… they were ready to go to landslide areas …they were ready to go to flood areas. camps and everywhere they were ready … but when they were asked to go to the airport, they were not willing. when we wanted them to enter some data from the declaration form from the airport, they didn’t even want to touch the declaration form …even though the form also comes 10 hours after the passenger has actually touched it. But still they did not want to touch it. Someone else has to scan and mail them to the other side of the room to get the data entered. So, you can see that they were so scared.”*

*(Director from Ministry of Health)*

*“So once lockdown was lifted in May, what we found was that because of these public health measures that have been enforced and recommended by the Government there was a lot of phobia… not only among the general public but also among health workers. they were reluctant to move out and start working”*

*(Representative from an international organization)*

*“For example, the consultant physicians and several others who were supposed to be working very closely in emergency situations were so reluctant to see the patients without ensuring the patient was free from COVID which is an impossible task in an emergency … I saw that the readiness of the health system was very poor at the beginning…. Now it is gradually picking up … up to now the readiness is something I am concerned about.”*

*(Director from Ministry of Health)*

*“Staff wants to kind of get protected from the patient, then they wanted to have a personal protective equipment … this was a huge challenge, because they were not freely available …the personal protective equipment were kind of misused at the early stage”*

*(Director from Ministry of Health)*

Even though the facilities increased with greater investments to accommodate more patients, the human resources did not increase leaving the existing work force exhausted.

*“The other thing is the exhaustion of the health staff because of that people were not well and could not cope up work with all the work, … the preventive health sector was the most affected as they had to do COVID monitoring, home quarantining, doing PCR and RAT antigen tests etc.”*

*(Deputy Regional Director of Health Services from Ministry of Health)*

While some healthcare workers showed a lack of readiness to provide services, there were other gaps in preparedness that were noted during this time. Sri Lanka had originally planned to conduct a parliamentary election in June 2020 but it had to be postponed to mid-August 2020 due to the COVID-19 pandemic. During elections, some government services are restricted according to election law prohibiting insinuations that such services are used for election propaganda. Therefore, some proactive government services were curtailed during this time, which further affected COVID-19 control.

*“Regular services were provided as much as possible till election time… And health systems were working… but the implementation was slowed down because of this election mode … because once you get into the declaration of election most government servants actually take a step back without being proactive.”*

*(Representative from an international organization)*

Initially, testing facilities available in the Ministry of Health was limited. There was a shortage of test kits and a dearth of places to carry out the tests. Testing facilities were slowly expanded in the country with purchase of needed equipment.

*“Initially it was only MRI (Medical Research Institute) doing PCR testing …then it was expanded slowly to hospitals in Kandy, Karapitiya and Anuradhapura and then Ragama, and then to other places in many general hospitals … at the beginning the testing facilities were limited. So, because of that, the samples had to be transported a very long distance. And then there’s a waiting list for testing. And because of that, the results were not immediately available.”*

*(Director from Ministry of Health)*

The Ministry of Health also faced barriers in training personnel to conduct laboratory testing during the pandemic. Initially, the Ministry decided to train many categories of employees in rapid antigen testing to ensure that a higher number of tests could be carried out within the country. However, the designated employees who routinely conduct diagnostic tests were unhappy with other groups of employees being trained to perform the test, as they felt that this was a skill that should be given only to people with their particular job role.

*“You know the rapid antigen test can be done by a trained laborer …but a certain category of people say that it should be done by them and no one else …these kinds of bottle necks should be eased out. If not in future also this can happen … trade unions involvement in this kind of activity should be handled in a very serious way…”*

*(Director from Ministry of Health)*

Access to data was another gap in the preparedness plan that was identified. Many departments under the Ministry of Health did not have access to data obtained from airports, isolation or quarantine facilities. They felt making decisions without data was dangerous and ineffective.

*“The issue is that the data is not in the form that will be available to a third party. That is one of the most important aspects to strengthen the response”*

*(Director from Ministry of Health)*

*“At present someone will stay at a government quarantine center for fourteen days but what is the risk of them coming to the community and spreading the disease. We don’t know. Because when they leave the quarantine center, we don’t do another PCR test on them… So, we don’t actually have any data on how many have got the disease after coming from a quarantine center back to their homes. In Sri Lanka, still this data is not available. We are taking those decisions without scientific data. That part is a major issue.”*

*(Director from Ministry of Health)*

### Theme 2: COVID-19 management

There were a number of strategies implemented to manage the spread of COVID-19 in Sri Lanka. Some of the strategies were based on research outcomes available on the effect of COVID-19 on vulnerable groups. The Ministry of Health also observed the steps taken by other countries to control the spread of COVID-19 and made calculated decisions based on their observations and findings.

#### Subtheme 1: Dual burden for the curative sector

The curative sector had to manage both COVID-19 patients and non-COVID patients. Hence, there was a compromise of services. For the non-COVID patients, issues were experienced for both inward patients and outpatients. For inward patients, space was a problem as priority was given to COVID-19 patients and the two types of patients could not be mixed. For outpatients, a triage system had to be developed and implemented.

*“Then the curative sector had to manage both COVID-19 and other non-COVID patients … so, there was a kind of compromising of certain services to a certain extent …for inward care again, there was the problem of the space, because we can’t keep both patients together … space was a problem in almost all hospitals”*

*(Deputy Director from Ministry of Health)*

*“Now in outpatient care there was a huge challenge whether patients coming into hospital are positive or not … then the a patient triage system was implemented, to identify whether patients were infected or not”*.

*(Deputy Regional Director of Health Services from Ministry of Health)*

#### Subtheme 2: Strategies to reduce transmission

To reduce transmission of COVID-19, a lockdown was implemented in densely populated areas where the risk of transmission was high. As many studies had found that COVID-19 resulted in fatalities in individuals with non-communicable diseases (NCDs), the Ministry of Health ensured that individuals with non-communicable diseases continued to receive their medication despite the lockdown regulations. The public who attended government clinics for prevention of NCDs were asked to register by phone with the institute and the medicines were distributed to ensure an uninterrupted supply. This was carried out under the supervision of the nearest Post Office. This was a strategy the Ministry of Health took to reduce the risk of fatalities amongst the public.

*“We tried our best to make sure that their co-morbidities are under control all the time wherever possible. That is how we tried to reduce complications despite the spread of COVID… we did this by visiting lockdown areas and distributing drugs to those who need. That is how we tried to assist the communities affected especially the vulnerable groups. In some situations, we also posted the medications to the people. Our morbidity and mortality were reduced to a great extent … not transmission but the number of severe cases and the number of deaths were reduced to a great extent after starting this process.”*

*(Director from Ministry of Health)*

Quarantining was another method that was used to reduce COVID-19 transmission within the country. All citizens and expatriates who had permission to enter Sri Lanka were brought back through repatriation flights and were quarantined either in government facilities at no cost or at a hotel with self-paid quarantine facilities. In addition, if someone from the community was diagnosed with COVID-19, the first contacts of this person had to be quarantined while the COVID-19 infected person was treated at the Infectious Diseases Hospital or any other designated institution.

*“So, mandatory quarantine was brought in. So, any person entering Sri Lanka had to go to this government quarantine center… So, through this actually we basically were able to prevent this COVID disease coming to our community… A few months back about fifty thousand people had registered to come. So, there was an issue…we did not have enough quarantine centers to put them. So, then the government took a policy to basically involve the private sector that means the hotels. They introduced the hotel quarantine… here people were able to pay and go and stay in hotel quarantine. Although it is run by the private sector, the access control and other control measures are done through the army.”*

*(Director from Ministry of Health)*

*“Then the government took a decision to quarantine the first contacts at their homes. That means home quarantine. But there were certain instances, all people didn’t have the facility to home quarantine. So, in those instances they were taken to government quarantine centers. Other than that, at present mainly the first contacts are home quarantined.”*

*(Director from Ministry of Health)*

#### Subtheme 3: Barriers to managing COVID-19

There were a number of barriers that healthcare workers encountered when managing the spread of the virus. Perceptions of the public, logistical barriers and lack of human resources were a few of the barriers that were identified by the interviewees.

The perceptions of the public on the virus were extremely negative. The public followed the guidelines laid down by the government. However, they feared the fact that health care workers visited their areas for COVID-19 related work, in the belief that the workers may be infected with the virus. Many people also did not declare their symptoms, fearing that they would have to be quarantined. There were a lot of negative messages through the media regarding the quarantine facilities and this discouraged people from receiving the treatment and management they required.

*“Basically, there was a resistance from the people… people thought that this quarantine was the huge harassment for them. basically mandatory quarantine for fourteen days then again another fourteen days of home quarantining, total twenty-eight days, when someone in the community was detected with COVID the media came… the police came … the inspectors came… COVID was feared like leprosy and became a stigmatized disease. Then the people basically did not want to come out with their disease condition. That is happening. They are not coming for PCR testing. They hide their symptoms…This resulted in the disease spread also.”*

*(Director from Ministry of Health)*

*“General public also had certain reservations about public health persons visiting their homes. Like midwives coming home. There was a lot of tension in the sense that they were not sure whether our health workers were affected and that they will bring the virus to their homes. So, there was a little bit of hesitancy”*

*(Representative from an international organization)*

The Ministry of Health experienced many logistical barriers. Organizing transport services for infected persons to quarantine centres and clearing intensive care beds for COVID-19 were extremely challenging logistical issues that had to be attended to during the management process. The interviewees also mentioned how exhausting the entire process has been for the people working to manage the effect of COVID-19.

*“Sending people to hospital… sending them back after discharging, these were all major operations that we had to undertake. It was highly challenging but because of a few people who were really committed to do it we managed. Now these people are also exhausted. We need more committed people for these kinds of activities.”*

*(Director from Ministry of Health)*

*“We had about 500 intensive care beds …we ear marked 146 beds for this activity to be specific, but when comes to the real utilization of intensive care beds it was another challenge, because we cannot keep intensive care beds vacant … if one person is there in the intensive care then we cannot keep COVID patients there … clearing beds was another very challenging part. At present we are managing by clearing around 25 beds for COVID management.”*

*(Director from Ministry of Health)*

There was also a lack of human resources. Although Sri Lanka has encountered and managed many crisis situations, COVID-19 stretched the human resources of the healthcare system. Unlike other crisis situations, the entire country needed to be monitored and therefore, it was not possible to mobilize human resources from one province to another. Furthermore, volunteer opportunities could not be encouraged under these circumstances because of the social distancing guidelines that were implemented by the Government.

*“But it was challenging to bring even one or two public health inspectors to the Colombo Municipality area because the services of these people are needed elsewhere also. As a result, the system did not permit to mobilize human resources from other areas …”*

*(Director from Ministry of Health)*

*“We had to monitor the entire geographic area of the island in different ways to make sure things are happening in a proper way… that was the major challenge…even if people wanted to help, they couldn’t come because of the social distancing guidelines.”*

*(Director from Ministry of Health)*

The pandemic also adversely affected the national economy. Due to the limited availability of emergency funds within the country, Sri Lanka was unable to cover the costs of the required interventions to control the spread of the virus. Expenses related to establishing and maintaining quarantine facilities, providing meals and other services to each person at these facilities, cost for disinfection activities, transport costs of patients to quarantine facilities were additional costs for the government. Construction of isolation rooms and conversion of outpatient service centers to COVID-19 wards were also tasks that required funding.

Sri Lanka received donations and loans to combat the virus. The WHO invested USD 5million to strengthen Sri Lanka’s response. The World Bank also provided a 3-year loan of USD 217 million, allocated to the Ministry of Health and the Ministry of Social Services.

*“More than fifteen-thousand rupees for one person when you consider the food and other things… Apart from these we have to spend on the salaries of the army, the facilities and everything… it was like around fifteen-thousand to twenty-thousand rupees for fourteen days per person. Then there is a transport cost as well… for example we will assume you are going from here to Kandakadu… the vehicles are going, the army guards are going… when returning also they have to transport all those people.”*

*(Director from Ministry of Health)*

*“The maintenance of the quarantine centres are also supported by this loan (World Bank loan)… from the electricity bills, the food, the equipment the furniture … the maintenance costs of 30–40 quarantine centers was managed by using this money…”*

*(Representative from an international organization)*

Polymerase Chain Reaction (PCR) and antigen testing, which were the diagnostic tests used in Sri Lanka, were other significant expenditures. In addition to the costs of these tests, the swab taking process, transportation and laboratory-related work were all additional expenses. Personal protective gear had to be provided for each healthcare worker carrying out tests. Furthermore, viral transport media had had to be purchased.

*“Assuming that the basic cost of a PCR test is six thousand rupees… ten thousand tests a day…you can imagine the amount of money we have to spend in one day… we had to buy PPE, test kits, the viral transport mediums, swabs and other type of sample taking items … “*

*(Director from Ministry of Health)*

The external funding was also used to strengthen contact tracing. When a patient is diagnosed with COVID-19 in Sri Lanka, Public Health Inspectors (PHI) identify and notify their contacts and ensure they are home quarantined. For this purpose and for other transportation needs, a portion of the World Bank loan was used to purchase vehicles for public health inspectors and public health midwives. The Ministry of Social Services used USD 89 million of the World Bank loan to provide money for vulnerable groups.

*“Ministry of Social Services used the money to support the cash transfers for elderly care program as well the cash transfers for this chronic kidney disease people… and to support the low-income families*.

*(Representative from an international organization)*

### Theme 3: Effects of COVID-19

COVID-19 has affected many areas of functioning in Sri Lanka, both positively and negatively. Many of these changes are likely to have long term repercussions in the country.

#### Subtheme 1: Negative effects

While the economic burden is an obvious disadvantage that arose due to COVID-19, there are other negative effects that this virus brought about such as monitoring other conditions and regular follow up of patients. As a result of lockdowns and social distancing guidelines, the management of other public health programmes were neglected. There is minimal focus on other public health activities as a result of the current situation as some personal were redeployed. Professional trainings are now being conducted virtually, and this may not be the most effective method to acquire new skills. If these trends continue, it will have a negative effect on the quality of life of people in Sri Lanka.

*“…there were other problems as well. For example, checking of blood pressure was not happening for some patients, even for a one-year period. And patients who needed to get laboratory investigations like patients having kidney disease. investigations were not done and they were just giving the drugs without checking the routine investigations.”*

*(Deputy Regional Director of Health Services from Ministry of Health)*

*“I don’t think other public health programmes have been implemented to the scale expected because with the indicators being mapped till 2025 and SDG indicators till 2030, we had given the targets… reduction of child infant mortality was also one … all those projects will have to definitely be rethought with this COVID situation… if COVID continues till 2021 there is going to be a huge impact on all public health programmes which includes nutrition, NCDs and also the non-health areas like education and environment … all those sectors are going to be affected. I think the entire SDG targets will have to be looked at again…”*

*(Representative from an international organization)*

*“You can’t have physical meetings…you can’t have trainings… except online. Online trainings work for some but for skilled trainings like what we do in the health sector, it’s a challenge. So, likewise all the programmes are affected due to this situation and we being the health sector, we don’t want to violate the guidelines that we developed… because if we violate our own guidelines then there is no way where the other departments and general public will have faith in our system.”*

*(Representative from an international organization)*

Another consequence of COVID-19 was a drop in the attendance at most health clinics and an overall decline in demand for health services, with some exceptions.

*“Two weeks into lockdown, mothers were asking for the vaccination. Because there was a demand created by the people… they knew the value of child vaccinations. Now likewise we did not see much of a demand from the general public regarding other services except for NCD medication.”*

*(Representative from an international organization)*

#### Subtheme 2: Positive effects

Some positive developments have taken place due to COVID-19. For instance, the capacity of laboratory services has been improved in terms of ability to perform a wider range and greater volume of investigations, and better equipment. One interviewee stated that there is also a reduction in acute respiratory infections and diarrhoeal diseases because of practices such as handwashing and wearing face masks.

*“During 2020 we have been able to strengthen the COVID response including laboratory strengthening … it is a part of our mandate …*. *4 million dollars plus closer to 5 million was actually invested to strengthen the Sri Lankan situation*… *I think the positive side is that we were able to strengthen laboratories from what we had …now be able to detect the viruses using PCR”*

*(Representative from an international organization)*

The development of quarantine facilities within the country has been expanded exponentially. Transport facilities for field health services also have been improved.

*“30-bed isolation facility at Infectious Disease Hospital (IDH) was newly constructed. 6 provincial hospitals were identified and upgraded to mini IDH to have a center for disease control in those hospitals… They used some funds to refurbish some of those hospitals to convert into isolation rooms and OPDs to patient centres in selected hospitals… for example in Warakapola and Matale…”*

*(Representative from an international organization)*

*“They purchased 930 motor cycles for the PHI… also the government procured 21 cabs for Medical Officers of Health (MOHs) … this was done to strengthen the mobility support at grassroot level. Now we’re in the process of procuring 1000 scootys [a two wheeled mode of transport] for PHMs …”*

*(Representative from an international organization)*

A feature especially during the second wave was the development of innovative solutions. Various individuals and organizations including the armed forces came up with novel sanitizer dispensers, remote communication systems to communicate with and monitor the condition of isolated patients.

*“There were some innovations as well. So, this is a positive thing during the second wave… there were a lot of innovations from other sectors as well like sanitizer machines and some remote communication systems”*

*(Deputy Regional Director of Health Services from Ministry of Health)*

Lastly, coordination among inter-governmental organizations and across many governmental sectors commenced or increased due to the pandemic. In addition, with increasing number of cases the public and other well-wishers also contributed significantly.

*“Without the army logistic support, definitely we would not have been able to manage this. For example, when we initially decided to quarantine the flights, at the time we only had the Hendala Leprosy hospital to quarantine…two wards were selected. But only one hundred persons could be kept there. Basically, with half a flight it will be filled. Then only the President instructed the army to put up these quarantine centres. Definitely without those quarantine centres we would not have been able to do this quarantine operation.”*

*(Director from Ministry of Health)*

*“Donations were received from in-country people as well as the foreign people. Now, I know many instances when we received calls asking whether the we want money and so on”*

*(Deputy Regional Director of Health Services from Ministry of Health)*

Sri Lanka is currently experiencing tremendous challenges as a result of the COVID-19, pandemic. Recent studies on pandemic response show that the earlier a country took active steps to control the spread of COVID-19, the more effective the strategies were in containing and slowing down the crisis [6, 7]. Although Sri Lanka took early steps to curb the spread of the virus during the first wave and, to a lesser extent, during the second wave, the virus has still adversely affected the economy, health system, education and wellbeing of citizens.

#### Health security preparedness

Sri Lanka had an emergency preparedness plan but the demand of COVID-19 was overwhelming and beyond the capacity the country had prepared for. A strengthened emergency preparedness plan is a need for many countries across the world. Many studies point out that mitigating stockouts of medical supplies such as PPE is an area that must be addressed in the emergency preparedness plans [8, 9]. Although Sri Lanka had an initial stock of PPE, there were other gaps within the preparedness plan that needed to be addressed.

Educating the public on emergency preparedness is an area that must be included in Sri Lanka’s emergency preparedness plan. It was evident that the public had difficulty adhering to guidelines put forward by the government during the pandemic. For instance, there were patients who absconded treatment or did not cooperate with contact tracing due to the public fear about the virus and the conditions in quarantine centers. Therefore, empowering the public on mitigating emergency situations is likely to reduce such issues in the future [10, 11].

Training and educating healthcare staff to work under high-risk conditions while ensuring personal safety has also been identified as an area that must be included in the emergency preparedness plans. For instance, a study conducted in Pakistan showed that 33% of healthcare workers showed a high level of anxiety to directly care for patients during the pandemic period [12]. Negative effects on mental wellbeing of healthcare workers have also been reported [13, 14]. As revealed by the interviewees in this study, some healthcare workers in Sri Lanka were reluctant to work in high-risk situations and displayed a high level of anxiety. This, in turn, caused delays in the delivery of required health services. Often, the fears and anxiety of family members further exacerbated the anxiety of the frontline workers regarding COVID-19 [15] similar to that observed among the healthcare workers in Sri Lanka. The concerns of family regarding the spread of the virus were affecting their productivity at work.

#### COVID-19 management

The management of non-communicable diseases to reduce mortality using evidence-based strategies [16–18] was implemented by healthcare workers in Sri Lanka. Interventions provided for sexual and reproductive health, maternal and child heath, and non-communicable diseases were negatively affected in multiple countries through lockdowns [17, 19, 20] based on the excerpts of the interviews we carried out, similar views were expressed. Mustafa et al (2022) highlight the need for establishing concrete plans to ensure health systems resilience for non-emergency health services [21]. The findings from this study point out that national authorities and partners—including academia, international organizations and donors—need to work together on health emergency planning to cater to population health needs and thereby improve available emergency and non-emergency health services. Therefore, Sri Lanka requires a system that pays equal focus to emergency and non-emergency health services during a pandemic situation.

The establishment of quarantining facilities within the country was another strategy used to reduce the rate of COVID-19 transmission in Sri Lanka. However, some interviewees were concerned that COVID-19 was being transmitted amongst the occupants in quarantine facilities. Studies show that such transmission occurs through asymptomatic patients at quarantine centers [22]. As suggested by the interviewees, it would be beneficial to systematically monitor the health of occupants after leaving the quarantine center to reduce potential risk of community transmission.

#### Changes in health system operations due to COVID-19

There were major negative effects of the pandemic that affected all aspects of society in Sri Lanka. Economic disruptions have impacted every community. Schools have been closed for almost the entirety of the pandemic, interrupting education programs for students of all ages. In the health sector, training programs, sexual and reproductive health, maternal and child health and other environmental health programs were neglected.

*Health system operations*. The pandemic has impacted the health system and its operations in both negative and positive ways. The aggressive spread of the disease demanding more focus on COVID-19 management in terms of human and other resources, hampered other activities like blood pressure monitoring of the patients, routine laboratory investigations of patients with chronic kidney disease etc. This has also increased the number of dropouts from clinics, the effect of which we may see long after the pandemic has been declared over. Similar findings have been reported from Northern Italy where emergency department visits for non-COVID-19 patients decreased with increased out-of-hospital mortality [23]. Comparable findings have been reported by others [24–26]. Although most studies have reported that online training of staff was a positive outcome, our interviewees felt that online training is likely to be ineffective due to other constraints such as online connectivity and lack of devices. All of the above were the negative effects of COVID-19 pandemic on the health system. The collective impact of all of these factors on sustainable SDG targets is yet to be reviewed.

There were indirect benefits that resulted from the pandemic. Laboratory facilities were upgraded in all major hospitals to conduct PCR testing. Hospitals, intensive care units and high dependency units were upgraded to accommodate the growing number of patients. Ventilators, high flow oxygen delivery systems and other equipment were provided by the government as well as through generous donations by well-wishers. Quarantine centers were established and today the country has sufficient quarantine capacity to meet any situation. The nature of the response to the pandemic has strengthened inter-sectoral coordination and action. In addition, given the travel restrictions, authorities resorted to online systems for communication. If maintained, these improvements will equip Sri Lanka to better respond to potential future waves of COVID-19 and other pandemic threats. Similar effects on the health system have been reported by other countries where the pandemic had acted as an eye opener as well as a driving force in bringing about improvements to the existing system [27]. There were reports of adaptation of health systems to meet the emergency; in some countries, ICU bed capacity was increased, adaptations in emergency and outpatient departments. There was expansion of many public health laboratories globally [28, 29].

### Economic effects of COVID-19

The pandemic contracted the global economy and the impact was particularly experienced by low- and middle-income countries, including Sri Lanka. A study conducted in four sub-Saharan African countries showed that 77% of the population (256 million individuals) had lost their income during the pandemic [30]. A large proportion of Sri Lanka’s working population belongs to the informal sector comprising daily wage earners [31]. Similar to the findings of Josephson and colleagues, many families in Sri Lanka lost their modes of income, especially those engaged in the tourism industry, due to the restrictions imposed by the government to control the spread of the virus. The impact of the pandemic on the informal sector has not been estimated as yet. The Sri Lankan government therefore, took steps to provide financial assistance, though a very small amount, to low-income families. However, the provision of relief packages given to the public in need maybe difficult to continue on a regular basis due to the severe strain on the economy.

### Limitations

This study is based on interviews with seven high ranking personnel directly involved in the control of the pandemic and who are familiar with IHR. The interviews were conducted in November/December 2021 about six months after the evolution of the third wave. It is likely that some of the interviewees may have forgotten some facts. It was difficult to interview healthcare personnel holding senior administrative grade posts during this time given their heavy workload. However, we believe that the ideas expressed by the interviewees are representative of the situation during the first and second waves of the epidemic in the country.

We were mindful of reflexivity where preconceived ideas about the area of study or the participants whom we are studying may affect the conduct of the study or the interpretation of the results. During analysis, the team assumed that their different backgrounds and experiences might influence the perspective, and subsequently, the analysis. As the analysis was conducted by the research assistant, an independent evaluator, the analysis is likely to have delivered unbiased efficient results.

## Conclusions

Although Sri Lanka had difficulties in managing the epidemic, pandemic preparedness as a result of IHR training helped in mitigating the spread of COVID-19 during the first and second waves of the pandemic. The report of the Joint External Evaluation was endorsed by the Ministry of Health and implemented; the Quarantine Unit of the Ministry of Health, the focal point for IHR, develop protocols and standards. However, the magnitude of the pandemic was too great and many lessons have been learned where improvement is required.

A larger proportion of the effects of the pandemic has influenced badly on the healthcare system exposing its weaknesses and highlighting where new systems and protocols should be established to handle future calamities. However, it has also had some positive influence by way of capacity building among health staff, improving awareness of infections and prevention methods among the general public, encouraging innovations and improved facilities such as transportation for field staff in order to carry out their duties effectively.

The economic impact of the pandemic in a LMIC such as Sri Lanka affected low-income families to a great extent; national budgets should consider having allocations for substantial social benefits during emergencies, particularly to low-income families as an integral part of the disaster preparedness plan. But, for Sri Lanka which is in a severe debt crisis, this would be a distant dream.
